# How family caregivers of persons with advanced cancer assist with upstream healthcare decision-making: A qualitative study

**DOI:** 10.1371/journal.pone.0212967

**Published:** 2019-03-13

**Authors:** J. Nicholas Dionne-Odom, Deborah Ejem, Rachel Wells, Amber E. Barnato, Richard A. Taylor, Gabrielle B. Rocque, Yasemin E. Turkman, Matthew Kenny, Nataliya V. Ivankova, Marie A. Bakitas, Michelle Y. Martin

**Affiliations:** 1 School of Nursing, University of Alabama at Birmingham, Birmingham, Alabama, United States of America; 2 School of Medicine, University of Alabama at Birmingham, Birmingham, Alabama, United States of America; 3 Geisel School of Medicine, Dartmouth College, Hanover, New Hampshire, United States of America; 4 School of Health Professions, University of Alabama at Birmingham, Birmingham, Alabama, United States of America; 5 Center for Innovation in Health Equity Research, University of Tennessee Health Science Center, Memphis, Tennessee, United States of America; Nord University, NORWAY

## Abstract

**Aims:**

Numerous healthcare decisions are faced by persons with advanced cancer from diagnosis to end-of-life. The family caregiver role in these decisions has focused on being a surrogate decision-maker, however, little is known about the caregiver’s role in supporting upstream patient decision-making. We aimed to describe the roles of family caregivers in assisting community-dwelling advanced cancer patients with healthcare decision-making across settings and contexts.

**Methods:**

Qualitative study using one-on-one, semi-structured interviews with community-dwelling persons with metastatic cancer (n = 18) and their family caregivers (n = 20) recruited from outpatient oncology clinics of a large tertiary care academic medical center, between October 2016 and October 2017. Transcribed interviews were analyzed using a thematic analysis approach.

**Findings:**

Caregivers averaged 56 years and were mostly female (95%), white (85%), and the patient’s partner/spouse (70%). Patients averaged 58 years and were mostly male (67%) in self-reported “fair” or “poor” health (50%) with genitourinary (33%), lung (17%), and hematologic (17%) cancers. Themes describing family member roles in supporting patients’ upstream healthcare decision-making were: 1) seeking information about the cancer, its trajectory, and treatments options; 2) ensuring family and healthcare clinicians have a common understanding of the patient’s treatment plan and condition; 3) facilitating discussions with patients about their values and the framing of their illness; 5) posing “what if” scenarios about current and potential future health states and treatments; 6) addressing collateral decisions (e.g., work arrangements) resulting from medical treatment choices; 6) originating healthcare-related decision points, including decisions about seeking emergency care; and 7) making healthcare decisions for patients who preferred to delegate healthcare decisions to their family caregivers.

**Conclusions:**

These findings highlight a previously unreported and understudied set of critical decision partnering roles that cancer family caregivers play in patient healthcare decision-making. Optimizing these roles may represent novel targets for early decision support interventions for family caregivers.

## Introduction

In 2018 alone, there were over 600,000 U.S. individuals who were in their last year of life due to an advanced stage cancer [1]. From the time one is diagnosed with an incurable, metastatic cancer to end-of-life, numerous healthcare decisions must be faced. These decisions include choices about cancer treatments and surgeries, locations of care and providers, transitions in care (e.g., hospice, assisted living), self-care activities, insurance coverage, advance care planning, and preferences for treatment and location of care at end of life [2–7]. Most research on serious illness decision-making has been guided by the two-actor paradigm of shared decision-making that focuses on the provider and the patient [8]. However, emerging research over the past decade suggests this view is too narrow in scope. In a national sample of over 5,200 newly-diagnosed cancer patients, 49.4% reported that they shared decisions equally with family members and 22.1% reported having elicited at least some input on decisions. Family members are often present and very active in treatment decision-making encounters, providing logistical assistance (e.g., transportation, physical assistance) and informational and emotional social support [6, 8].

Furthermore, involvement of family members in decisions has been associated with positive patient outcomes, including patient satisfaction and adherence to medical treatments [9, 10]. Conversely, family caregivers who are not involved in helping patients make decisions may heighten the patient’s distress and lead to the receipt of care and treatments inconsistent with the patient’s values and preferences of their patients [6, 8, 11, 12]. While interventions have been developed and rested to enhance surrogate decision-making (both prospectively[13–19] and at end of life[20–29]), far less is known about how to best support family roles when they partner with patients in upstream healthcare decision-making.

Evidence suggests that these family members are performing critical functions in particular settings and contexts where patient decision-making is taking place, such as in provider encounters about treatment choices, [6] chronic illness treatment decisions, [8] and discussions about cancer clinical trial enrollment [4]. However, little has been reported on the roles families play across settings and contexts in cancer. Understanding this broad picture may facilitate the development of enhanced upstream decision support that goes beyond the traditional patient-provider actors in shared decision making and targets a broader set of family caregiver decision partnering skills that apply across settings and contexts. To further explore this area, the purpose of this study was to identify the decision-making roles of family caregivers of community-dwelling patients with advanced cancer.

## Methods

Following standards of the COnsolidated criteria for REporting Qualitative research (COREQ) guidelines, [30] this was a qualitative, descriptive study conducted as part of a formative evaluation of upstream telehealth intervention for rural-dwelling advanced cancer family caregivers [31]. One-on-one semi-structured interviews were conducted either in-person or over the phone with family caregivers and their care recipients with advanced cancer to inquire about the kind of support that families offered patients when faced with healthcare decisions due to having advanced cancer. Because little has been reported in this area, a purposive sample of caregivers and patients was chosen with a variety of advanced cancer types.

### Ethics statement

This study was approved by the Institutional Review Board at the University of Alabama at Birmingham. All study participants signed informed consent and were compensated $20. Precautions were taken to minimize the risk of loss of participant confidentiality including de-identifying all participant forms, digitally-recorded interviews, and transcriptions and storing them as encrypted files on the principal investigator’s (PI’s) password protected computer and keeping informed consent forms in a locked file cabinet in the PI’s locked office.

### Participants, recruitment, and data collection

Patients with advanced cancer and their family caregivers were recruited between October 2016 and October 2017 from outpatient oncology clinics at a large academic tertiary care hospital. Patients were eligible if they were 21 years or older and had Stage III/IV brain, breast, gynecologic, gastrointestinal, genitourinary, lung, melanoma, and hematologic cancers. Exclusion criteria included having medical record documentation of severe mental illness (schizophrenia, bipolar disorder), active substance abuse, suicidal ideation, or dementia. As part of the formative evaluation portion of the study, [31] patients also had to reside in a U.S. Census designated rural zip code. The patient did not have to participate in order for the caregiver to participate in the study. A family member or friend who was 21 years of age or older was eligible for the study as a “family caregiver” if they or the patient identified them as an individual who the patient knows well and who assists with their day-to-day medical care. Exclusion criteria included self-endorsing severe mental illness (schizophrenia, bipolar disorder), active substance abuse, suicidal ideation, or dementia. Targeted sample size was estimated to be between 12–20 patients and 12–20 family caregivers based on qualitative guidelines to attain saturation [32]. To identify potential patient and family caregiver participants, the electronic medical record was screened weekly for patients appearing to meet eligibility criteria with an outpatient appointment in the following 2 weeks. Patients and family members were approached by the study’s project manager in the waiting room prior to the visit with their oncologist to introduce the study, confirm eligibility, and acquire informed consent. Consenting individuals were then scheduled for a one-on-one in-person at the hospital or telephone interview lasting up to 1 hour with the principal investigator (JND-O), using a semi-structured interviewing guide (see **Box 1** for sample interview questions) developed in consultation with the study’s multidisciplinary co-investigative team (DE, RAT, GBR, NVI, MAB, MYM), representing sociology, nursing, medicine, clinical psychology, oncology, mixed methods research, palliative care, and healthcare disparities research and pilot tested with its members for clarity.

**Box 1. Interview questions**

1. After being diagnosed with cancer, what kinds of healthcare decisions do patients and families face?
2. In what ways have you/has your family member been involved in facing any decisions that you/your loved one have faced?
3. What have you/has your family member done to help make these decisions?
4. How do you think you/your family member will assist with healthcare decisions you might face in the future?

### Data analysis

Caregiver and patient interviews were digitally-recorded and transcribed by a professional transcription service (Landmark Associates), uploaded for analysis into Atlas.ti software, and reviewed for accuracy. Data were analyzed using a thematic analysis approach consistent with the steps outlined by Braun and Clarke [33, 34] immediately subsequent to transcription of the first interview and continuing through November 2018. First, to facilitate immersion and familiarity with the data, all transcripts were read independently by the analysis team (JND-O, DE, RW, MB). Second, line-by-line coding began with open coding [35] of all transcripts conducted by two members of the team, the principal investigator (JND-O), a board certified hospice and palliative care advanced practice nurse and experienced qualitative researcher, and a study co-investigator (DE), a medical sociologist and dyadic spiritual coping researcher. Third, open codes were combined into preliminary overarching themes by the two open coding analysts using within and across case matrices to facilitate raw data comparison [36]. Finally, preliminary themes and their corresponding textual support and initial descriptions were presented to other members of the study team (RW, MB) for final refinement by assessing “fit” of the themes to the raw data and the degree to which the collection of themes represented a complete picture of the entire data corpus.

#### Trustworthiness

Strategies to uphold rigor of this qualitative study followed criteria outlined by Lincoln and Guba (i.e., credibility, transferability, dependability, and confirmability) [37]. A diverse analysis team with differing professional backgrounds and areas of expertise to promote reflexivity [38] convened every 2 to 4 weeks throughout the analysis period to discuss the coding and themes and the data analysis process itself. In addition, an audit trail was kept of all steps of the analysis, including matrices of codes, themes and raw data for thick descriptions and reflective memos were used to facilitate analysis team discussions [36].

## Findings

### Sample characteristics

In total, 38 individuals completed in-depth semi-structured interviews: 18 patients and 20 family caregivers (**Table 1**). Patients were on average 58 years of age and were mostly male (67%), white (83%), married or living with a partner (78%), and Protestant (89%). The majority had a high school education or less (60%). A wide range of cancer types were represented with half the sample self-endorsing that they were in “poor” or “fair” health and half in “good”, “very good”, or “excellent” health. Family caregivers were mostly the spouse or partner of the patient (70%) and on average 56 years of age. Most were female (95%), white (85%), married or living with a partner (90%), and Protestant (85%). The majority had some college or more of education (55%) and worked part or full-time (55%). On average, caregivers had been providing care for more than 2 years and the majority provided care 4 or more days a week (75%) for 3 or more hours per day (55%).

**Table 1:** Participant characteristics.

	Patients		Family Caregivers	
	No.	%	No.	%
**Age, Mean (SD), Range**	58.0 (10.4), 40–84		56.0 (12.8), 28–77	
**Gender**				
Female	6	33.3	19	95.0
Male	12	66.7	1	5.0
**Race**				
White	15	83.3	17	85.0
African-American/Black	3	16.7	3	15.0
**Marital Status**				
Married or living with partner	14	77.8	18	90.0
Divorced or separated	3	16.7	1	5.0
Single	1	5.6	0	0
Widowed	0	0.0	1	5.0
**Education**				
Masters	1	5.6	2	10.0
College graduate	2	11.1	2	10.0
Some college	3	16.7	7	35.0
Vocational	2	10.2	0	0
High school	9	50.0	7	35.0
Some high school	2	10.2	2	10.0
**Socioeconomic Status (Total Household Income)**				
<$30,000	6	33.3	8	40.0
$30,000-$49,999	5	27.8	3	15.0
>$50,000	7	38.9	8	40.0
Rather not say	0	0	1	5.0
**Religion**				
Protestant	16	88.9	17	85.0
No religious affiliation	1	5.6	1	5.0
Other	1	5.6	2	10.0
**Employment Status**				
Employed full time	5	27.8	7	35.0
Employed part time	1	5.6	4	20.0
Retired or Homemaker	3	16.7	7	35.0
Unemployed	9	50.0	2	10.0
**Advanced cancer type**				
Genitourinary	6	33.3	-	-
Lung	3	16.7	-	-
Hematologic	3	16.7	-	-
Brain	2	11.1	-	-
Gastrointestinal	2	11.1	-	-
Breast	1	5.6	-	-
Gynecologic	1	5.6	-	-
**My health is…**				
Poor or Fair	9	50.0	6	30.0
Good	4	22.2	8	40.0
Very Good or Excellent	5	27.8	6	30.0
**Relationship to patient (This person is my…)**				
Spouse/partner	-	-	14	70.0
Parent	-	-	1	5.0
Child	-	-	1	5.0
Other family member	-	-	2	10.0
Sibling	-	-	1	5.0
Friend/Neighbor	-	-	1	5.0
**Months as a caregiver, Mean (SD), Range**	-	-	27.1 (34.9), 1–135
**Days per week providing care**				
<1	-	-	5	25.0
4–5	-	-	3	15.0
6	-	-	1	5.0
Everyday	-	-	11	55.0
**Hours per day providing care**				
<1	-	-	4	20.0
1–2	-	-	5	25.0
3–4	-	-	2	10.0
5–6	-	-	1	5.0
7–8	-	-	2	10.0
>8	-	-	6	30.0

### Qualitative results

Analysis of family caregiver and patient participants interviews yielded 7 main themes representing key roles played by family members when assisting and supporting patients with their healthcare decision-making. These roles are presented as themes below and additional example quotes supporting each theme are shown in **Table 2**.

**Table 2:** Family caregiver roles in supporting the healthcare decision-making of persons with cancer.

Theme/Role	Example Quotes
1) Cancer and treatment information seeker	*“With me*, *to me information is strength*. *…since he has been diagnosed there’s not been an entire week that’s went by that I’ve not got on the Internet and search different trials or different breakthroughs or different new clinical studies or anything that’s been coming out with—that has to do with his diagnosis*. *…bein’ able to know what our options are*, *because once the shoe drops*, *you don’t have time to make up your mind*. *…You don’t have time to research it then*. *You have to be ahead of the game a little bit*. *You have to know what hospitals and what locations are offering what…Which trials have been showing the best promise*, *… you have to try to have an idea of what your choices may be before you have to even make the choice*.*”* (CG 009)
	*“It’s always helpful to get some information no matter what the situation is*. *You can deal much better with things if you know what you don’t want*.*”* (CG 022)
2) Shared understanding facilitator	*“…that's so I can ask questions that she may not think of or make sure she has a clear understandin' everything that's goin' on…”* (CG 028)
	*“I try to impress upon him how important it is to tell me every little side effect*, *everything he deals*, *so that I can communicate to the doctors*.*”* (CG 023)
	*“…they'll keep up with everything that the doctor says and make sure that you follow it*, *I think that's one of the most important things*.*”* (PT 020)
	*“I’m the one who’s got the kids out here freaking out*. *His family who doesn’t understand—they’re elderly*. *They don’t understand*. *My elderly parents who don’t understand why all this fell apart*. *That’s the caregiver who has to explain to everybody who’s freaked out about why the plans didn’t go as they were supposed to*.*”* (CG 014)
3) Values and illness framer discussant	*“Well*, *we've pretty much discussed her wishes should she have a turn for the worse or something like that*. *We've gone over what her desires are for if she passes or something like that*. *As far as her cancer in this moment*, *I mean*, *she's a fighter and she wants to live*. *She's in no shape*, *form*, *or fashion is thinkin' about givin' up or anything…”* (CG 028)
	*“…she said “That’s not the way you’ve got to look here*. *You’ve got look at it like it’s another challenge in life*. *You’ve got to look at it like it’s a challenge*. *You’ve got to learn how to step up and do things*, *like taking care of yourself*. *Looking out for your disabilities now*. *Whatever it takes to make your life a bit better”* (PT 023)
4) “What if” scenario poser	*“I would like to be able to gather information and maybe get some ideas of which direction I need to go should scenario one happen*, *or which way I should go should scenario two happen*, *that kind of thing*.*”* (CG 019)
	*“You got to start the conversation with trying to*, *‘Well*, *what would you like to do if this happens*? *What’s your plan for if this happens*?*’”* (CG 032)
5) Collateral decisions manager	*“He just sat down and looked at the different options with me*, *financially*, *and with him workin’ when he could—how he could rearrange his schedule with his job*, *to be able to go to the treatments with me and make sure that he was able to be there for all my doctors’ appointments and stuff like that*.*”* (PT 019)
	*“He had gotten fired from his job a few months prior*. *I believe it was probably because of the tumor*, *because he was havin’ headaches all the time before he knew*. *At the point that he got diagnosed*, *he did not have medical insurance*. *…We’d been datin’ forever anyway and talked about marriage anyway*. *We got married*. *I don’t regret that decision*. *That was a decision*. *So that he could have surgery and they could at least see him*, *we got married*. *Of course*, *then he was covered under my insurance*.*”* (CG 009)
	*“She’s wantin’ to sell the house*, *get somethin’ smaller*. *…We’ve kinda discussed it there a little bit*. *That’s just kinda her sayin’ she can’t take care o’ the place if somethin’ happened to me permanently*.*”* (PT 014)
6) Decision point originator	*“…I was the one who said*, *“You’re going in to the hospital*. *We’re going back to the doctor*. *This is not normal*. *You’ve got a fever*.*” He would lay there two or three days chilling*, *and spiking a temp*, *and taking Advil*. *I had to be the one to decide…”* (CG 014)
	*“…the hard decisions that I had to make with Mom*, *tellin’ ‘em*, *no*, *we’re not doin’ no more radiation for a week’s worth o’ life*, *no*.*”* (CG 029)
7) Delegated decision maker	*“I make all the decisions*. *Because he wasn’t in a place to make any of ‘em*. *… I had to make the decisions about the bills*, *about the house*, *about my job*. *Keeping his job informed*. *Letting them know*. *… I had to do everything*, *because he wasn’t capable*.*”* (CG 014)
	*“He leaves all the decisions up to me to make*. *That’s the way it’s been*.*”* (CG 016)
	*“He leaves it basically up to me and my opinion*. *…I think he relies on me a hundred percent*, *probably*.*”* (CG 020)
	*“They might say this or this or this*, *you know*, *[NAME OF CAREGIVER] be the one probably decides what we’re gonna do*.*”* (PT 003)

#### Cancer and treatment information seeker

Most patient (PT) and family caregiver (CG) participants described how family members would seek out, gather, and elicit information pertaining to the cancer diagnosis, its assessment including diagnostic and lab tests, and any proposed or potential treatments. Caregivers sought information from various sources including healthcare professionals (e.g., doctors and nurses), family and friends, books, the internet, and research studies. As the second quote in Table 2 for this theme expresses, this gathering of information appeared to represent a form of coping and advanced preparation for potential future decisions. As another participant described, “…you got to have this stuff already set in place so that all you have to do is push the button to release” (CG 019). Some indicated that this preparatory information gathering was necessary because once faced with the actual situation, there is not enough time to collect needed information. Patient participants made similar comments about this aspect of their family member’s role, particularly in the setting of meeting with doctors where they reported their family members often asking questions to clarify the cancers, its treatments, and tests: “…we got three ears to listen to what the doctor is telling us…That helps a lot. [caregivers] will keep up with everything that the doctor says…” (PT 020).

#### Shared understanding facilitator

Most patient and caregiver participants spoke about the ways that family caregivers interfaced with healthcare professionals, family, and friends and even patient themselves in order to communicate information to facilitate decision-making processes. Family members reported often communicating information to providers in order to fill in missing information that might not be reported by patients. Caregivers would also pose questions to doctors in order to indirectly help clarify the situation for patients who they felt might not be completely understanding the situation: “I couldn’t get him [THE PATIENT] to understand that the only way he was gonna be cured was with a bone marrow transplant. When we came back to Dr. —, I started then to get Dr.—to talk more about the bone marrow transplant. …Every time he would talk, I would think, “Okay, [PATIENT’S NAME] understands a little bit more.” (CG 014).

#### Values and illness framer discussant

Many participants talked about the role caregivers had in initiating and facilitating discussions pertaining to the patients’ values and how the illness was framed within the story of their lives, which ultimately had an impact on healthcare choices. Caregivers often influenced how patients framed their view of the illness: “You’ve got to learn how to step up and do things, like taking care of yourself. Looking out for your disabilities now. Whatever it takes to make your life a bit better” (PT 023). As the quote indicates, family caregivers would often encourage a positive reframing of the illness. In addition to helping patients reframe their current situation, they also facilitated conversations about prospective decision at end-of-life: “We've pretty much discussed her wishes should she have a turn for the worse or something like that. We've gone over what her desires are for if she passes or something like that” (CG 028).

#### “What if” scenario poser

Many participants described family members taking on the role of asking ‘what if’ during the course of cancer treatment. By asking ‘what if’, family members appeared to explore and compare various outcomes of pursing several courses of action given different health situations. Additionally, in introducing ‘what if’ scenarios, family members created a space to speculate on how the family members or the patients themselves would want a specific set of health scenarios handled. While some participants used specific ‘if this’ terminology to explore potential scenarios stemming from the cancer or cancer treatments, others forecasted multiple potential situations. CG 032 describes this patient-led speculation on potential situations in the following quote from Table 2, “You got to start the conversation with trying to, ‘Well, what would you like to do if this happens? What’s your plan for […] if this happens?’” whereas a quote from CG 019 reflects more introspective speculation, “I would like to be able to gather information and maybe get some ideas of which direction I need to go should scenario one happen, or which way I should go should scenario two happen, that kind of thing.”

#### Collateral decisions manager

Many participants described instances where family members dealt with unanticipated, non-medical decisions resulting as a consequence of choices made in treatment decision-making. Family members may help manage decisions related to the ripple effect of cancer treatment decisions, such as a need for a more flexible work schedule: “He just sat down and looked at the different options with me, financially, and with him workin’ when he could—how he could rearrange his schedule with his job, to be able to go to the treatments with me and make sure that he was able to be there for all my doctors’ appointments and stuff like that” (PT 019). One participant (CG 009) spoke about the acceleration of marriage plans as a decision stemming from treatment needs: “At the point that he got diagnosed, he did not have medical insurance. …We’d been datin’ forever anyway and talked about marriage anyway. We got married. I don’t regret that decision. That was a decision. So that he could have surgery and they could at least see him, we got married. Of course, then he was covered under my insurance.” In the above collateral decision management, family members were motivated by patient needs (i.e. transport/support at appointments, insurance), but other participants describe other collateral decisions management motivated by caregiver considerations. Patient 014 spoke about the caregiver contemplating the size of their home in the context of his illness, “She’s wantin’ to sell the house, get somethin’ smaller. …We’ve kinda discussed it there a little bit. That’s just kinda her sayin’ she can’t take care o’ the place if somethin’ happened to me permanently.” Both family member and patient participants talked about the management of collateral decisions often noting the influence of healthcare decisions on these non-healthcare decisions.

#### Decision point originator

Several participants spoke in detail about specific instances when family members were the ones that identified a critical moment in a situation requiring consideration of potential alternative courses of action. These decision points included decisions to seek out emergency assistance, as was noted by CG 014 in Table 2. The other quote in the Table by CG 029 highlighted other decision points, namely moments when it was time to decide on a different treatment or treatment approach. As described by another participant, “I had to tell that doctor ‘no’, we’re not doing no more radiation for a week’s worth of life.” Some caregivers talked about moments when they felt that seeking a different provider or healthcare institution was necessary: “I wondered should we stay around here in Birmingham or should we take him—try to get him in Kentucky doin’ a clinical trial right know that’s havin’ really great results or try to get him in at Duke or take him to UCLA” (CG 009).

#### Delegated decision maker

When asked about how family members were involved with the patients’ decision-making, several participants emphasized that nearly all healthcare decisions were made by the family caregiver. In this delegated decision-making role, participants described scenarios where the family member was responsible for most decision-making tasks, as found in quotes by CG 016, “He leaves all the decisions up to me to make. That’s the way it’s been.” and CG 020, “He leaves it basically up to me and my opinion. …I think he relies on me a hundred percent, probably.” While many participants did not describe a formal discussion of delegation, one caregiver emphasized the de facto nature of the role of delegated decision maker: “I make all the decisions. Because he wasn’t in a place to make any of ‘em. … I had to make the decisions about the bills, about the house, about my job. Keeping his job informed. Letting them know. … I had to do everything, because he wasn’t capable.” (CG 014). Patients often noted the role of their family members as primary decision maker, even in shared decision making with providers, as demonstrated in the following patient quote, “They [providers] might say this or this or this, you know, [CAREGIVER NAME] be the one probably decides what we’re gonna do.” (PT 003).

## Discussion

We conducted a qualitative study of 20 family caregivers and 18 of their care recipients with advanced metastatic cancers aimed that explored the roles family caregivers assume in assisting patients with treatment and other healthcare decisions. Our analysis identified 7 roles undertaken by these family members, including: cancer and treatment information seeker, shared understanding facilitator, decision point originator, values and illness framer discussant, “what if” scenario poser, collateral decisions manager, and delegated decision maker. These results are consistent with a growing literature on family involvement in serious illness decision-making [6, 8, 39, 40] and extends prior insights by highlighting an understudied set of critical decision partnering roles by cancer family caregivers across contexts and care settings.

The propensity for family caregivers to seek out cancer and treatment option information is a role well documented in the literature, particularly in the context of triadic (i.e., provider-patient-caregiver) treatment decision encounters [6, 39, 41]. Patients commonly have cognitive limitations due to chemotherapy or the cancer itself that interfere with their ability to seek out, process, and remember complex information necessary to understand their cancer diagnosis and the available treatment choices [42–44]. Family members in our study appeared to compensate for this limitation by becoming a source of informational social support, which may, as some evidence suggests, alleviate some of the patient’s burden and distress [4, 45]. However, the nature and degree to which this assistance affects patients’ choices is largely unknown and warrants further investigation. In addition, the severity of the potential burden of the informational social support role on the family member is also unknown, though many tested cancer caregiver interventions include psychoeducation and others forms of information provision to help support this role [46].

Three other decision partnering roles identified in this analysis related in part to the information seeking role were being a facilitator of shared understanding of the decision-making situation, being a values and illness framer discussant, and posing “what if” hypothetical scenarios. Participants described various efforts to facilitate communication and information exchange between themselves and the patient and other pertinent parties (i.e., healthcare clinicians, other family and friends) in order to promote a shared and informed understanding of events, which has been referred to by others as distributed cognition or “shared mind” [4, 47]. This type of communication, especially when targeted at values and illness framing, appeared intended in part to influence the process of decision-making or the selection of particular choices; hence future research might explore the social influence strategies employed by caregivers to impact healthcare decision-making (e.g., framing effects). A number of caregivers also described thinking about and discussing “what if” situations with their care recipients in order to help mentally forecast what the implications would be and what would happen if certain decisions were made. Future research should examine the functions of this mental forecasting, including potentially helping patients and families discriminate pros and cons among choices currently being considered and promoting proactive coping and preparedness through planning about a potential future decision point.

Among other novel findings from our analysis was the role family caregivers play in originating decision points and managing collateral decisions. Participants discussed moments when a family caregiver felt a situation required a decision about whether there needed to be a change in course of action (e.g., seeking emergency care). Caregivers also managed collateral decisions, or decisions that arose as a result of cancer treatment decisions that themselves were not necessarily healthcare related. The implications and effects of advanced cancer are rarely contained to health and may extend to employment, finances, childcare, and other ‘collateral’ aspects of day-to-day life. Facing collateral decisions such as rearranging work obligations and managing finances is a fairly common challenge reported in studies of cancer caregivers [48, 49] and a recent systematic review noted that studies of caregivers often report how one decision often leads to another [40]. To our knowledge, decision support for these roles has not been developed.

The majority of individuals with cancer desire to have their family members involved to some degree in their healthcare decisions [11]. Our finding where several patients expressed that all of their healthcare and other decisions are delegated to their family caregivers likely represents one end of a spectrum of the type and extent of involvement patients desire from their family caregivers in assistance with healthcare decision-making. Patients may vary in the extent to which they wish to be autonomous in their healthcare decisions such that if a patient has a preference for high autonomy, then receiving unsolicited support from family members may be perceived negatively as controlling and condescending [4, 50]. On the other hand, if patients have a high preference for family and more distributed decision-making (e.g., Asian and African-American culture), then lack of shared decision-making from family may be negatively perceived [51]. Ultimately, more work is needed in diverse and global populations as a recent systematic review of family involvement in patient decision-making did not identify any studies outside of North America and Europe [8]. Relatedly, there is a lack of ways to measure the magnitude and positivity/negativity of the influence that family caregiver decision partnering roles have on patient decision-making and overall patient quality of life [40].

There are several limitations to note for this study. First, this was a qualitative study and hence results are not generalizable but rather transferable and hypothesis generating, providing an in-depth understanding of participant’s views and experiences. Relatedly, the demographics of the sample were homogenous in several respects, as participants were all rural-dwelling and mostly white, Protestant, and in a spousal relationship. Future work will benefit from larger, more representative samples and include populations representing greater global diversity. Second, our results cannot speak to differences in perceptions between caregivers and patients of the roles that family caregivers play in healthcare decision-making. It would be worth further investigating both normative and descriptive differences in patient versus caregiver perceptions of the decision partnering role as has been done by others [39, 45]. Third, this study did not focus on any particular healthcare decision and several studies have suggested that there may be distinct differences in how family caregivers are involved with particular decisions, such as decisions about major surgery [52] or chemotherapy clinical trial participation [4]. Future research should further specify family decision partnering roles in these particular situations in order to inform the development of the most appropriate decision support. Ultimately, the primary strength of this study is the identification of conceptual areas that can be used to base components of novel interventions to enhance the decision partnering skills of cancer family caregivers.

In conclusion, healthcare decision-making in cancer has often focused on the relationship between patients and their physicians. Findings from our study however, suggest that family caregivers are involved in critical decision partnering roles that likely significantly influence the decision-making processes and actual choices made by patients. This is concerning given that caregivers often do not feel included or supported in the healthcare decision-making process [40]. Hence, we recommend that clinicians inquire about and support the decision partnering roles of family caregivers for their care recipients with advanced cancer. Furthermore, we believe it is imperative that future research further explore these decision partnering roles and develop and test decision support interventions that extend beyond the patient to their family caregivers who support them in coping with serious cancers. Given the lack of effective interventions to support the decision partnering role of families [40], we believe this is a priority next step to guiding the development of healthcare system and government-level policies and educational initiatives.

## References

[1] Islami F, Goding Sauer A, Miller KD, Siegel RL, Fedewa SA, Jacobs EJ, et al Proportion and number of cancer cases and deaths attributable to potentially modifiable risk factors in the United States. CA Cancer J Clin. 2018;68(1):31–54. Epub 2017/11/21. 10.3322/caac.21440 . 29160902

[2] Bakitas M, Kryworuchko J, Matlock D, Volandes A. Palliative Medicine and Decision Science: The critical need for a shared agenda to foster informed patient choice in serious illness. Journal of Palliative Medicine. 2011;14(10):1109–16. Epub Sep 6 2011. PubMed Central PMCID: PMC21895453. 10.1089/jpm.2011.0032 21895453 PMC3236099

[3] Rini C, Jandorf L, Goldsmith RE, Manne SL, Harpaz N, Itzkowitz SH. Interpersonal influences on patients' surgical decision making: the role of close others. J Behav Med. 2011;34(5):396–407. Epub 2011/02/10. 10.1007/s10865-011-9323-y 21308408 PMC3113663

[4] Krieger JL, Krok-Schoen JL, Dailey PM, Palmer-Wackerly AL, Schoenberg N, Paskett ED, et al Distributed Cognition in Cancer Treatment Decision Making: An Application of the DECIDE Decision-Making Styles Typology. Qual Health Res. 2017;27(8):1146–59. Epub 2016/05/12. 10.1177/1049732316645321 . 27179018

[5] Lee JE, Shin DW, Cho J, Yang HK, Kim SY, Yoo HS, et al Caregiver burden, patients' self-perceived burden, and preference for palliative care among cancer patients and caregivers. Psychooncology. 2015 10.1002/pon.3827 . 25920720

[6] Laidsaar-Powell RC, Butow PN, Bu S, Charles C, Gafni A, Lam WW, et al Physician-patient-companion communication and decision-making: a systematic review of triadic medical consultations. Patient Educ Couns. 2013;91(1):3–13. Epub 2013/01/17. 10.1016/j.pec.2012.11.007 . 23332193

[7] Petrillo LA, McMahan RD, Tang V, Dohan D, Sudore RL. Older Adult and Surrogate Perspectives on Serious, Difficult, and Important Medical Decisions. J Am Geriatr Soc. 2018;66(8):1515–23. Epub 2018/07/04. 10.1111/jgs.15426 29972594 PMC6167167

[8] Lamore K, Montalescot L, Untas A. Treatment decision-making in chronic diseases: What are the family members' roles, needs and attitudes? A systematic review. Patient Educ Couns. 2017 Epub 2017/08/14. 10.1016/j.pec.2017.08.003 . 28838630

[9] Wolff JL, Roter DL. Hidden in plain sight: medical visit companions as a resource for vulnerable older adults. Arch Intern Med. 2008;168(13):1409–15. 10.1001/archinte.168.13.1409 . 18625921

[10] DiMatteo MR. Social support and patient adherence to medical treatment: a meta-analysis. Health Psychol. 2004;23(2):207–18. 10.1037/0278-6133.23.2.207 . 15008666

[11] Hobbs GS, Landrum MB, Arora NK, Ganz PA, van Ryn M, Weeks JC, et al The role of families in decisions regarding cancer treatments. Cancer. 2015;121(7):1079–87. Epub 2015/02/23. 10.1002/cncr.29064 25708952 PMC4368490

[12] Clayman ML, Roter D, Wissow LS, Bandeen-Roche K. Autonomy-related behaviors of patient companions and their effect on decision-making activity in geriatric primary care visits. Soc Sci Med. 2005;60(7):1583–91. 10.1016/j.socscimed.2004.08.004 . 15652689

[13] Ditto PH, Danks JH, Smucker WD, Bookwala J, Coppola KM, Dresser R, et al Advance directives as acts of communication: a randomized controlled trial. Arch Intern Med. 2001;161(3):421–30. . 11176768 10.1001/archinte.161.3.421

[14] Detering KM, Hancock AD, Reade MC, Silvester W. The impact of advance care planning on end of life care in elderly patients: randomised controlled trial. BMJ. 2010;340:c1345 Epub 2010/03/23. 10.1136/bmj.c1345 20332506 PMC2844949

[15] Song MK, Ward SE, Fine JP, Hanson LC, Lin FC, Hladik GA, et al Advance care planning and end-of-life decision making in dialysis: a randomized controlled trial targeting patients and their surrogates. Am J Kidney Dis. 2015;66(5):813–22. Epub 2015/06/30. 10.1053/j.ajkd.2015.05.018 . 26141307 PMC5972830

[16] Sulmasy DP, Hughes MT, Yenokyan G, Kub J, Terry PB, Astrow AB, et al The Trial of Ascertaining Individual Preferences for Loved Ones' Role in End-of-Life Decisions (TAILORED) Study: A Randomized Controlled Trial to Improve Surrogate Decision Making. J Pain Symptom Manage. 2017;54(4):455–65. Epub 2017/07/14. 10.1016/j.jpainsymman.2017.07.004 28712987 PMC5632104

[17] Bravo G, Sene M, Arcand M, Hérault É. Effects of advance care planning on confidence in surrogates' ability to make healthcare decisions consistent with older adults' wishes: Findings from a randomized controlled trial. Patient Educ Couns. 2018;101(7):1256–61. Epub 2018/02/10. 10.1016/j.pec.2018.02.005 . 29452728

[18] Green MJ, Van Scoy LJ, Foy AJ, Stewart RR, Sampath R, Schubart JR, et al A Randomized Controlled Trial of Strategies to Improve Family Members' Preparedness for Surrogate Decision-Making. Am J Hosp Palliat Care. 2017:1049909117744554. Epub 2017/01/01. 10.1177/1049909117744554 . 29186982 PMC5943708

[19] Hanson LC, Carey TS, Caprio AJ, Lee TJ, Ersek M, Garrett J, et al Improving decision-making for feeding options in advanced dementia: a randomized, controlled trial. J Am Geriatr Soc. 2011;59(11):2009–16. Epub 2011/09/15. 10.1111/j.1532-5415.2011.03629.x 22091750 PMC3227016

[20] White DB, Angus DC, Shields AM, Buddadhumaruk P, Pidro C, Paner C, et al A Randomized Trial of a Family-Support Intervention in Intensive Care Units. N Engl J Med. 2018;378(25):2365–75. Epub 2018/05/23. 10.1056/NEJMoa1802637 . 29791247

[21] Daly BJ, Douglas SL, O'Toole E, Gordon NH, Hejal R, Peerless J, et al Effectiveness trial of an intensive communication structure for families of long-stay ICU patients. Chest. 2010;138(6):1340–8. Epub 2010/06/24. 10.1378/chest.10-0292 20576734 PMC2998207

[22] Carson SS, Cox CE, Wallenstein S, Hanson LC, Danis M, Tulsky JA, et al Effect of Palliative Care-Led Meetings for Families of Patients With Chronic Critical Illness: A Randomized Clinical Trial. JAMA. 2016;316(1):51–62. 10.1001/jama.2016.8474 27380343 PMC5538801

[23] Curtis JR, Back AL, Ford DW, Downey L, Shannon SE, Doorenbos AZ, et al Effect of communication skills training for residents and nurse practitioners on quality of communication with patients with serious illness: a randomized trial. JAMA. 2013;310(21):2271–81. 10.1001/jama.2013.282081 24302090 PMC4310457

[24] Curtis JR, Nielsen EL, Treece PD, Downey L, Dotolo D, Shannon SE, et al Effect of a quality-improvement intervention on end-of-life care in the intensive care unit: a randomized trial. Am J Respir Crit Care Med. 2011;183(3):348–55. Epub 2010/09/10. 10.1164/rccm.201006-1004OC 20833820 PMC3056230

[25] Curtis JR, Treece PD, Nielsen EL, Gold J, Ciechanowski PS, Shannon SE, et al Randomized Trial of Communication Facilitators to Reduce Family Distress and Intensity of End-of-Life Care. Am J Respir Crit Care Med. 2016;193(2):154–62. 10.1164/rccm.201505-0900OC 26378963 PMC4731711

[26] Lee Char SJ, Evans LR, Malvar GL, White DB. A randomized trial of two methods to disclose prognosis to surrogate decision makers in intensive care units. Am J Respir Crit Care Med. 2010;182(7):905–9. Epub 2010/06/10. 10.1164/rccm.201002-0262OC 20538959 PMC2970862

[27] Lautrette A, Darmon M, Megarbane B, Joly LM, Chevret S, Adrie C, et al A communication strategy and brochure for relatives of patients dying in the ICU. N Engl J Med. 2007;356(5):469–78. 10.1056/NEJMoa063446 . 17267907

[28] Azoulay E, Forel JM, Vinatier I, Truillet R, Renault A, Valade S, et al Questions to improve family-staff communication in the ICU: a randomized controlled trial. Intensive Care Med. 2018;44(11):1879–87. Epub 2018/10/21. 10.1007/s00134-018-5423-2 . 30374690

[29] Aslakson R, Cheng J, Vollenweider D, Galusca D, Smith TJ, Pronovost PJ. Evidence-based palliative care in the intensive care unit: a systematic review of interventions. J Palliat Med. 2014;17(2):219–35. 10.1089/jpm.2013.0409 24517300 PMC3924791

[30] Tong A, Sainsbury P, Craig J. Consolidated criteria for reporting qualitative research (COREQ): a 32-item checklist for interviews and focus groups. Int J Qual Health Care. 2007;19(6):349–57. Epub 2007/09/14. 10.1093/intqhc/mzm042 . 17872937

[31] Dionne-Odom JN, Taylor R, Rocque G, Chambless C, Ramsey T, Azuero A, et al Adapting an Early Palliative Care Intervention to Family Caregivers of Persons With Advanced Cancer in the Rural Deep South: A Qualitative Formative Evaluation. J Pain Symptom Manage. 2018;55(6):1519–30. Epub 2018/02/21. 10.1016/j.jpainsymman.2018.02.009 29474939 PMC5951755

[32] Patton M. Qualitative research and evaluation methods: Integrating theory and practice. Thousand Oaks, CA: Sage; 2014.

[33] Braun V, Clarke V. Using thematic analysis in psychology. Qualitative Research in Psychology. 2006;3(2):77–101.

[34] Vaismoradi M, Turunen H, Bondas T. Content analysis and thematic analysis: Implications for conducting a qualitative descriptive study. Nursing and Health Sciences. 2013;15:398–405. 10.1111/nhs.12048 23480423

[35] Saldana J. The Coding Manual for Qualitative Researchers. Washington DC: Sage; 2013.

[36] Miles M, Huberman A, Saldana J. Qualitative Data Analysis: A Methods Sourcebook. Third ed Thousand Oakes: Sage Publications, Inc.; 2014.

[37] Lincoln Y, Guba E. Naturalistic Inquiry. Newbury Park, CA: Sage Publications; 1985.

[38] Barry CA, Britten N, Barber N, Bradley C, Stevenson F. Using reflexivity to optimize teamwork in qualitative research. Qual Health Res. 1999;9(1):26–44. 10.1177/104973299129121677 . 10558357

[39] LeBlanc TW, Bloom N, Wolf SP, Lowman SG, Pollak KI, Steinhauser KE, et al Triadic treatment decision-making in advanced cancer: a pilot study of the roles and perceptions of patients, caregivers, and oncologists. Support Care Cancer. 2018;26(4):1197–205. Epub 2017/11/04. 10.1007/s00520-017-3942-y . 29101469

[40] Garvelink MM, Ngangue PA, Adekpedjou R, Diouf NT, Goh L, Blair L, et al A Synthesis Of Knowledge About Caregiver Decision Making Finds Gaps In Support For Those Who Care For Aging Loved Ones. Health Aff (Millwood). 2016;35(4):619–26. 10.1377/hlthaff.2015.1375 . 27044961

[41] Goldsmith DJ, Miller GA. Conceptualizing how couples talk about cancer. Health Commun. 2014;29(1):51–63. Epub 2013/02/26. 10.1080/10410236.2012.717215 . 23442190

[42] Hlubocky FJ, Sachs GA, Larson ER, Nimeiri HS, Cella D, Wroblewski KE, et al Do Patients With Advanced Cancer Have the Ability to Make Informed Decisions for Participation in Phase I Clinical Trials? J Clin Oncol. 2018;36(24):2483–91. Epub 2018/07/09. 10.1200/JCO.2017.73.3592 29985748 PMC6097830

[43] Vega JN, Dumas J, Newhouse PA. Cognitive Effects of Chemotherapy and Cancer-Related Treatments in Older Adults. Am J Geriatr Psychiatry. 2017;25(12):1415–26. Epub 2017/04/06. 10.1016/j.jagp.2017.04.001 28495470 PMC5630507

[44] Shin DW, Cho J, Roter DL, Kim SY, Park JH, Yang HK, et al Patient's Cognitive Function and Attitudes towards Family Involvement in Cancer Treatment Decision Making: A Patient-Family Caregiver Dyadic Analysis. Cancer Res Treat. 2018;50(3):681–90. Epub 2017/07/04. 10.4143/crt.2017.201 28701031 PMC6056983

[45] Shin DW, Cho J, Roter DL, Kim SY, Yang HK, Park K, et al Attitudes Toward Family Involvement in Cancer Treatment Decision Making: The Perspectives of Patients, Family Caregivers, and Their Oncologists. Psychooncology. 2017;26(6):770–8. Epub 2016/09/15. 10.1002/pon.4226 . 27437905

[46] Ferrell B, Wittenberg E. A review of family caregiving intervention trials in oncology. CA Cancer J Clin. 2017 Epub 2017/03/20. 10.3322/caac.21396 . 28319263

[47] Epstein RM. Whole mind and shared mind in clinical decision-making. Patient Educ Couns. 2013;90(2):200–6. Epub 2012/08/11. 10.1016/j.pec.2012.06.035 . 22884938

[48] National Alliance for Caregiving. Cancer Caregiving in the U.S.: An Intense, Episodic, and Challenging Care Experience. Bethesda, MD: National Alliance for Caregiving, 2016.

[49] Merrill Lynch. The Journey of Caregiving: Honor, Responsibility and Financial Complexity. Charlotte, NC: 2017.

[50] Hauke D, Reiter-Theil S, Hoster E, Hiddemann W, Winkler EC. The role of relatives in decisions concerning life-prolonging treatment in patients with end-stage malignant disorders: informants, advocates or surrogate decision-makers? Ann Oncol. 2011;22(12):2667–74. Epub 2011/03/21. 10.1093/annonc/mdr019 . 21427061

[51] Cain CL, Surbone A, Elk R, Kagawa-Singer M. Culture and Palliative Care: Preferences, Communication, Meaning, and Mutual Decision Making. J Pain Symptom Manage. 2018;55(5):1408–19. Epub 2018/01/31. 10.1016/j.jpainsymman.2018.01.007 . 29366913

[52] Hall DE, Morrison P, Nikolajski C, Fine M, Arnold R, Zickmund SL. Informed consent for inguinal herniorrhaphy and cholecystectomy: describing how patients make decisions to have surgery. Am J Surg. 2012;204(5):619–25. Epub 2012/09/01. 10.1016/j.amjsurg.2012.07.020 . 22944389 PMC7224355

